# The Levels and Influencing Factors of Compassion Fatigue among New Nurses during the COVID-19 Pandemic

**DOI:** 10.1155/2023/4362841

**Published:** 2023-07-11

**Authors:** Li Zeng, Dong Liu, Xiaoli Liang, Lan Li, Yihang Peng, Man Jin, Wanqing Xie, Jialin Wang

**Affiliations:** ^1^Sichuan Nursing Vocational College, 173 Longdu South Road, Longquanyi District, Chengdu, Sichuan 610100, China; ^2^School of Pharmacy, Chengdu Medical College, 783 Xindu Avenue, Xindu District, Chengdu, Sichuan 610500, China; ^3^The Third People's Hospital of Chengdu, No. 82 Qinglong Street, Qingyang District, Chengdu, Sichuan 610014, China; ^4^West China Dental Hospital of Sichuan University, No. 14 Renmin South Road, Wuhou District, Chengdu, Sichuan 610000, China; ^5^College of Nursing, Chengdu University of Traditional Chinese Medicine, No. 1166 Liutai Road, Wenjiang District, Chengdu, Sichuan 611137, China

## Abstract

**Aim:**

To investigate the levels and influencing factors of compassion fatigue among new nurses during the COVID-19 pandemic.

**Background:**

New nurses are often unable to cope with the escalating nursing problems during the COVID-19 epidemic because of their lack of work experience, which may reduce their level of compassion satisfaction and cause compassion fatigue. However, there is little research on compassion fatigue and compassion satisfaction among new nurses during the COVID-19 epidemic.

**Methods:**

This was a cross-sectional study. From June to October 2021, 520 new nurses were selected from eight designated hospital for treatment of COVID-19 in China for electronic survey. Questions elicited social-demographics, work-related information, lifestyle factors, and the Chinese version Professional Quality of Life Scale.

**Results:**

The scores of compassion satisfaction, burnout, and secondary traumatic stress of new nurses were 31.85 ± 6.18, 27.94 ± 5.04, and 27.17 ± 4.87, respectively. In addition, regression analysis showed that nurses' compassion satisfaction, burnout, and secondary traumatic stress were affected by social-demographics, work-related information, and lifestyle factors.

**Conclusions:**

During the COVID-19 epidemic, compassion satisfaction, burnout, and secondary traumatic stress of new nurses were at a moderate level, which were relatively affected by work-related information and lifestyle, and had little correlation with social-demographics. *Implications for Nursing Management*. Hospital administrators can promote new nurses' compassion satisfaction and alleviate their compassion fatigue by improving job satisfaction, encouraging smoking cessation, arranging work hours reasonably, ensuring sleep hours, reducing workplace violence, and strengthening the reporting of violence incidents.

## 1. Introduction

Since COVID-19 was first reported in Wuhan, China, at the end of 2019, it has rapidly swept the world and become a major health issue worldwide [1]. Due to the continuous development, spread, and repetition of COVID-19 epidemic, more nurses are required to maintain good physical and mental conditions to face patients and provide effective care for patients [2, 3]. Unfortunately, the loss of nursing talents and the lack of nursing team personnel have always been an important challenge for hospital administrators around the world, especially during the COVID-19 epidemic [4]. Gong et al. [5] believed that recruiting and training new nurses is one of the valid ways to supplement nursing talents and expand the nursing team. However, during the COVID-19 epidemic, new nurses (defined as nurses who have been engaged in clinical nursing work for less than 3 years) who have just entered the workplace have received little attention [3]. In fact, new nurses are often unable to deal with the escalating nursing problems during the epidemic because of lack of work experience and slow adaptation to clinical work, which may cause them to have various mental health problems, such as compassion fatigue, and may eventually lead to their resignation [6, 7]. Therefore, in order to reduce the impact of COVID-19 epidemic on new nurses and further stabilize the nursing team, it is necessary to clarify the prevalence and influencing factors of compassion satisfaction and compassion fatigue of new nurses during the COVID-19 epidemic.

## 2. Background

### 2.1. Compassion Fatigue

During the COVID-19 epidemic, the nurses had high work intensity and heavy work tasks and were frequently exposed to traumatic situations such as virus infection and serious illness of patients, which increased the work pressure of nurses and easily led to frequent mental health problems, such as compassion fatigue [2, 8]. Compassion fatigue is often used as a substitute term for the results of various occupational stresses, which refer to emotional exhaustion caused by long-term in-depth contact with patients, excessive empathy, and exposure to occupational stress [9]. Specifically, compassion fatigue included burnout and secondary traumatic stress [10]. Among them, burnout was described as a sense of frustration and fatigue caused by long-term work pressure, and secondary traumatic stress was considered as an occupational hazard to caregivers caused by repeated exposure to patients' traumatic experiences [11, 12]. Coetzee and Laschinger [13] believed that compassion fatigue might lead to nurses' apathy, unresponsiveness, lack of judgment, and even collapse in nursing work, which would then affect their work efficiency and care level. Moreover, Xie et al. [14] pointed out that young and inexperienced nurses were more prone to burnout and secondary traumatic stress than senior nurses, and the level of compassion fatigue was higher.

### 2.2. Compassion Satisfaction

In contrast to the negative impact of compassion fatigue on nurses' physical and mental health, compassion satisfaction is considered to be the sense of achievement and satisfaction that nurses get by alleviating patients' pain through clinical nursing work and nursing behavior, which is a positive response [15]. Compassion satisfaction is closely related to the sense of personal achievement, which originates from helping others to survive traumatic events as caregivers and obtaining positive support from others [14, 16]. Some studies showed that compassion satisfaction could provide nurses with work motivation, enhance their insight, strengthen their sense of faith, and alleviate their compassion fatigue, so as to promote nurses to show an optimistic and positive work attitude and high-level work efficiency and strong willingness to stay, which is conducive to improving the overall nursing quality [17, 18]. Moreover, Wang et al. [15] believed that nurses with rich work experience had a higher level of compassion satisfaction, and on the contrary, new nurses had a lower level of compassion satisfaction, which may be affected by their lack of clinical experience.

Although many previous studies have investigated the level of compassion satisfaction and compassion fatigue of nurses, there are few studies on new nurses, especially during the COVID-19 epidemic. In view of the frequent occurrence and serious consequences of compassion fatigue during the COVID-19 epidemic, in order to reduce the impact of compassion fatigue on new nurses' physical and mental health and help hospital administrators retain nursing staff, our research objective is to describe the current situation of compassion satisfaction and compassion fatigue of new nurses during the COVID-19 epidemic. The specific aims of this study were to (1) evaluate the prevalence of compassion satisfaction, burnout, and secondary traumatic stress of new nurses during the COVID-19 epidemic and (2) clarify the impact of social-demographics, work-related information, and lifestyle on new nurses' compassion satisfaction, burnout, and secondary traumatic stress.

## 3. Methods

### 3.1. Aims

This study aimed to investigate the levels and influencing factors of compassion fatigue among new nurses during the COVID-19 pandemic.

### 3.2. Design

A cross-sectional survey design was used.

### 3.3. Participants

From June 2021 to October 2021, participants were selected from eight Class A tertiary general hospitals designated to treat patients with COVID-19 in Sichuan Province, China, by convenient sampling. The participants work in different departments, including internal medicine, surgery, obstetrics, pediatrics, emergency, ICU, and infection department. The inclusion criteria were as follows: (a) registered nurses, (b) completed prejob training and engaged in clinical nursing for 6 months to 3 years, and (c) informed consent and voluntary participation in the study. Exclusion criteria were as follows: (a) nonformal staff of the investigated hospital and (b) those who are not on duty due to various reasons (such as health problems and advanced studies).

### 3.4. Data Collection

Before the investigation, the hospital administrators were contacted, and the investigation permission was obtained. The electronic questionnaire was distributed through WeChat and QQ groups, and the electronic questionnaire contained unified guidelines to introduce the purpose, variables, and questionnaire filling methods of this survey in detail. All items were set as required, and the same IP address could only be filled in once. The respondents were informed that the questionnaire was completed anonymously and voluntarily, and the completion and submission of the questionnaire was deemed as informed consent. The sample size calculation formula *N* *=* *Z*_1*−a/*2_^2^*p* (1−*p*)/*d*^2^ was used, where *Z*_1*−a*/2_ = 1.96 (at 5% type 1 error *P* < 0.05), *d* represented the allowable error, 0.05 was taken in this study, and *P* was based on a similar previous study, 22% [12]. According to the formula, the sample size was 264. Considering that there may be problems such as missing items in the questionnaire, the sample size was expanded by 20%, and it was concluded that at least 317 nurses need to be investigated. A total of 558 questionnaires were distributed, and after excluding 38 invalid questionnaires, a total of 520 questionnaires were collected, with an effective recovery rate of 93.19%. The STROBE checklist was used to report findings.

### 3.5. Measures

#### 3.5.1. Social-Demographics, Work-Related Information, and Lifestyle Questionnaire

The self-designed questionnaire was used to collect new nurses' social-demographics (age, gender, education level, and marital status), work-related information (shift predominantly worked, work hours per day, workplace violence experience, and job satisfaction), and lifestyle (sleep hours per day, smoking, and alcohol).

#### 3.5.2. The Chinese Version Professional Quality of Life Scale (ProQOL-CN)

ProQOL-CN, translated and revised by Chen and Wang [19] of the original ProQOL, developed by Stamm [10], was used. The ProQOL-CN includes three subscales: compassion satisfaction, burnout, and secondary traumatic stress, which are used to independently measure the level of compassion satisfaction, burnout, and secondary traumatic stress of nurses. Each subscale includes 10 items, a total of 30 items. The 5-point Likert scale is adopted, and the scores from “never” to “always” are 1–5, respectively, of which 1, 4, 15, 17, and 29 are reverse scoring items. The higher the score, the higher the level of compassion satisfaction, burnout, and secondary traumatic stress. It was found that ProQOL-CN has appropriate reliability and validity in Chinese nurse, and the scores of each subscale ≤22 are low level, 23–41 are moderate level, and ≥42 are high level [8, 19]. In this study, the Cronbach's *α* of compassion satisfaction, burnout, and secondary traumatic stress were 0.907, 0.782, and 0.790.

## 4. Data Analysis

All analyses were performed using SPSS version 26.0, and *P* < 0.05 was considered statistically significant (two-sided). Descriptive statistics were used to present the new nurses' social-demographics, work-related information, lifestyle, compassion satisfaction, burnout, and secondary traumatic stress. Independent *t*-tests were used to analyse the differences in gender, education level, main shifts, work hours per day, workplace violence experience, sleep hours per day, smoking, and alcohol. Study variables were compared among marital status and job satisfaction groups using one-way ANOVA. Multiple linear regression analysis models were performed to identify the influencing factors of compassion satisfaction, burnout, and secondary traumatic stress.

## 5. Ethical Consideration

The principles of anonymity and informed consent were strictly followed throughout the study, and this study has been approved by the Ethics Committee of Chengdu University of Traditional Chinese Medicine (number: 2020-KL084).

## 6. Results

### 6.1. Participant Characteristics

Of the 520 new nurses, 12.31% were males and 87.69% were females, with the average age of 23.64 ± 1.39. 40.19% of new nurses were single. The majority of new nurses had bachelor degree or above (68.27%), were on day shift (61.35%), worked hours per day >8 h (68.27%), and slept hours per day >7 h (66.54%). More than half of new nurses had workplace violence experience (60.96%) and thought their job satisfaction was average (53.46%). Only 4.04% of new nurses smoked and only 7.31% drank alcohol (Table 1).

#### 6.1.1. The Level of Compassion Satisfaction, Burnout, and Secondary Traumatic Stress

The scores of compassion satisfaction, burnout, and secondary traumatic stress of new nurses were 31.85 ± 6.18, 27.94 ± 5.04, and 27.17 ± 4.87, respectively. The low and moderate prevalence rates of compassion satisfaction were 93.65%, the moderate and high prevalence rates of burnout were 85.38%, and the moderate and high prevalence rates of secondary traumatic stress were 83.46% (Table 2).

#### 6.1.2. The Factors Associated with Compassion Satisfaction, Burnout, and Secondary Traumatic Stress

Independent t-tests and one-way ANOVA showed that new nurses, who worked day shift, had high job satisfaction, slept >7 h per day, did not smoke and drank alcohol, and had higher scores of compassion satisfaction, while new nurses who were single, night shift, worked >8 h per day, had low job satisfaction, slept ≤7 h per day, and experienced workplace violence, and had higher burnout scores, while new nurses who worked >8 h per day and experienced workplace violence had higher secondary traumatic stress scores (Table 1).

Multiple linear regression analysis showed that job satisfaction, sleep hours per day, smoking, and workplace violence experience were the influencing factors of compassion satisfaction of new nurses, which explained 22.1% of the total variance, while job satisfaction, sleep hours per day, and work hours per day were the influencing factors of new nurses' burnout, which explained 38.3% of the total variance, while workplace violence experience and work hours per day were the influencing factors of new nurses' secondary traumatic stress, which explained 2.1% of the total variance (Table 3).

## 7. Discussion

For nurses, compassion fatigue is an occupational hazard, providing compassion to others is the primary premise, and indirect contact with traumatic events is the “cost of caring” [15, 20]. Compassion satisfaction is a positive consequence of providing care services for the injured, which is conducive to improving nurses' work efficiency and nursing service quality [13]. The results of this study showed that during the COVID-19 epidemic, new nurses scored 31.85 ± 6.18 on compassion satisfaction, 27.94 ± 5.04 on burnout, 27.17 ± 4.87 on secondary traumatic stress, and the low and moderate prevalence rates of compassion satisfaction were 93.65%, the moderate and high prevalence rates of burnout were 85.38%, and the moderate and high prevalence rates of secondary traumatic stress were 83.46%. Compared with previous studies in Chinese oncology nurses [21], our findings were similar to their level of compassion satisfaction, but burnout and secondary traumatic stress were significantly higher. Furthermore, compared with a systematic review including 79 studies in various countries, new nurses in our study had a lower prevalence of compassion satisfaction and a higher prevalence of burnout and secondary traumatic stress [12]. This difference may be explained by the differences in different work fields, working years, cultural backgrounds, social environments, and individual characteristics of nurses. In addition, compared with Zhuang et al.'s [8] research on compassion fatigue of nurses under the COVID-19 epidemic, the level of compassion satisfaction of nurses in this study is lower, while the level of burnout and secondary traumatic stress is higher, which may be related to the fact that the respondents of this study are new nurses. New nurses have short working years and lack of work experience, so they cannot quickly adapt to and adjust their working conditions in the face of the COVID-19 epidemic, a public health emergency, prone to negative psychology or emotions. Therefore, based on the existing data, health institutions should pay more attention to how to help new nurses improve compassion satisfaction and alleviate compassion fatigue during the COVID-19 epidemic.

### 7.1. The Factors Associated with Compassion Satisfaction

Our research suggested that during the COVID-19 epidemic, higher job satisfaction and sleep hours per day >7 h had a positive impact on the compassion satisfaction level of new nurses. This is consistent with the findings of previous studies, which believed that nurses with higher job satisfaction and sufficient sleep time had stronger adaptability and recovery ability and could more appreciate their own value from the intense and cumbersome nursing work during the epidemic, thus promoting compassion satisfaction [8, 22]. In addition, our study showed that smoking and workplace violence experience had a negative impact on the compassion satisfaction level of new nurses. Itzhaki et al. [23] proposed that workplace violence would increase nurses' work stress and thus reduce nurses' compassion satisfaction. During the COVID-19 epidemic, most of the new nurses may suffer workplace violence due to lack of nursing experience and insufficient adaptability, which will increase their psychological burden and is not conducive to their compassion satisfaction. Moreover, Wang et al. [15] reported that smoking could affect nurses' compassion satisfaction as a negative factor, which is consistent with this study. Therefore, improving job satisfaction, ensuring sleep time, reducing the prevalence of workplace violence, and urging smoking cessation may be important means to support new nurses' compassion satisfaction during the COVID-19 epidemic.

### 7.2. The Factors Associated with Burnout

The current research results showed that new nurses with higher job satisfaction and sleep hours per day >7 h had lower burnout level, and new nurses with work hours per day >8 h had higher burnout level during the COVID-19 epidemic. In such a special environment as the COVID-19, the higher the job satisfaction of nurses, the more they can get achievement in the process of caring for patients, which provides motivation for their nursing work, strengthens their determination to face difficulties, and reduces the level of burnout [22]. Moreover, some studies suggested that scientific and reasonable working arrangement helped nurses to devote themselves to nursing care more wholeheartedly, while adequate sleep played a vital role in the rapid recovery of nurses after work, especially during the COVID-19 epidemic [1, 24]. Hence, promoting job satisfaction, limiting working hours to manageable levels, and ensuring sleep may help prevent new nurses from burnout during the COVID-19 epidemic.

### 7.3. The Factors Associated with Secondary Traumatic Stress

This study revealed that workplace violence experience and work hours per day >8 h were the positive factors of secondary traumatic stress of new nurses during the COVID-19 epidemic, which is consistent with the study of Okoli et al. [25] and Falatah and Alhalal [26]. On the one hand, during the COVID-19 epidemic, various violent acts against medical and health care personnel have increased, and nurses, who have the most contact with patients, also face the highest risk of violent attacks, especially new nurses with insufficient nursing experience, while nurses suffering from workplace violence had a higher level of secondary traumatic stress [18, 25, 27]. On the other hand, the normalization of the prevention and control of the COVID-19 epidemic have led to the extension of nurses' working hours and an increase in their workload, which has become the main cause of nurses' work pressure, and may directly affect their mental health and increase the risk of secondary traumatic stress [26, 28]. Thus, it is necessary to strengthen the attention to the mental health of new nurses, establish and improve the reporting system of violent incidents, and reasonably arrange the working time and workload to prevent the secondary traumatic stress of new nurses during the COVID-19 epidemic.

## 8. Limitations

There are some limitations in this study. First, the cross-sectional survey cannot explore the causal relationship between various factors. Second, convenience sampling may affect the universality of results. Third, we followed the principle of voluntariness in the investigation, and the questionnaires were all self-reported, which may lead to some bias in the research results. Finally, when investigating the workplace violence experience of new nurses, we did not distinguish the type and frequency of violence, and the interpretation rate of factors to secondary traumatic stress of new nurses was low, which may need to be explored in future studies.

## 9. Implications for Nursing Management

Medical and health organizations need to improve their understanding of compassion satisfaction and compassion fatigue of new nurses and develop targeted strategies, especially during the COVID-19 epidemic. On the one hand, they need to improve the job satisfaction through some specific means, such as allowing nurses to participate in shift scheduling, improving nurses' autonomy, and reasonably arranging workload, so as to promote compassion satisfaction and reduce the risk of compassion fatigue. On the other hand, encouraging smoking cessation, reasonably arranging working hours, ensuring rest time, reducing workplace violence, and strengthening the reporting after violent incidents can all improve compassion satisfaction of new nurses and alleviate their compassion fatigue.

## 10. Conclusion

Although there are some limitations in the study design, this study also provides some important information about compassion satisfaction, burnout, and secondary traumatic stress of new nurses during the COVID-19 epidemic. The results revealed that during the COVID-19 epidemic, compassion satisfaction, burnout, and secondary traumatic stress of new nurses were at a moderate level, which was relatively affected by work-related information and lifestyle, and had little correlation with social-demographics. Among them, job satisfaction and sleep hours per day are the factors that promote the increase of compassion satisfaction and the decrease of burnout, smoking is the negative factor that affects compassion satisfaction, workplace violence experience is the factor that promotes the decrease of compassion satisfaction and the increase of secondary traumatic stress, and work hours per day is the positive factor of burnout and secondary traumatic stress. These findings may have guiding significance for nursing managers to formulate work-related policies and practice to improve the physical and mental health of new nurses during the COVID-19 epidemic.

## Figures and Tables

**Table 1:** Demographic characteristics and scores of compassion satisfaction, burnout, and secondary traumatic stress of new nurses.

Variables	*N* (%)	Compassion satisfaction	Burnout	Secondary traumatic stress
		Mean (SD)	Mean (SD)	Mean (SD)
*Gender*				
Male	64 (12.31)	31.42 (7.04)	28.52 (5.17)	27.75 (6.01)
Female	456 (87.69)	31.91 (6.56)	27.86 (5.02)	27.09 (4.69)
*t*		0.350	0.944	1.043
*P*		0.555	0.332	0.308

*Education level*				
Associate degree or less	165 (31.73)	32.09 (6.71)	27.55 (5.24)	27.05 (5.18)
Bachelor degree or above	355 (68.27)	31.74 (5.92)	28.12 (4.94)	27.22 (4.73)
*t*		0.367	1.454	0.143
*P*		0.545	0.229	0.705

*Marital status*				
Married	115 (22.12)	32.24 (6.29)	27.97 (4.98)	27.57 (4.90)
Member of an unmarried couple	196 (37.69)	32.05 (5.59)	27.28 (4.38)	26.90 (4.48)
Single	209 (40.19)	31.45 (6.63)	28.55 (5.58)	27.20 (5.21)
*F*		0.769	3.237	0.687
*P*		0.464	**0.040**	0.504

*Shift predominantly worked*				
Day shift	319 (61.35)	32.47 (6.07)	27.42 (5.04)	27.15 (5.00)
Night shift	201 (38.65)	30.86 (6.24)	28.77 (4.95)	27.19 (4.68)
*t*		8.516	8.883	0.010
*P*		**0.004**	**0.003**	0.921

*Work hours per day*				
≤8 h	165 (31.73)	32.31 (6.49)	26.59 (4.97)	26.45 (5.09)
>8 h	355 (68.27)	31.64 (6.03)	28.57 (4.96)	27.50 (4.74)
*t*		1.334	17.851	5.302
*P*		0.249	**≤0.001**	**0.022**

*Workplace violence experience*				
Yes	317 (60.96)	31.72 (6.34)	28.60 (5.16)	27.68 (4.88)
No	203 (39.04)	32.05 (5.94)	26.92 (4.69)	26.37 (4.77)
*t*		0.363	14.143	9.064
*P*		0.547	**≤0.001**	**0.003**

*Job satisfaction*				
Very dissatisfied	27 (5.19)	25.44 (7.77)	34.67 (5.85)	28.07 (6.84)
Dissatisfied	82 (15.77)	28.38 (5.56)	32.04 (3.92)	27.84 (5.06)
Average	278 (53.46)	31.64 (5.34)	28.04 (3.85)	27.20 (4.71)
Satisfied	129 (24.81)	35.73 (5.30)	24.75 (3.95)	26.45 (4.64)
Very satisfied	4 (0.77)	35.75 (7.04)	23.81 (5.91)	26.25 (2.06)
*F*		33.670	74.568	1.382
*P*		**≤0.001**	**≤0.001**	0.239

*Sleep hours per day*				
≤7 h	174 (33.46)	30.71 (6.74)	29.37 (5.20)	27.66 (5.26)
>7 h	346 (66.54)	32.42 (5.81)	27.22 (4.81)	26.92 (4.66)
*t*		8.991	21.928	2.691
*P*		**0.003**	**≤0.001**	0.101

*Smoking*				
Yes	21 (4.04)	24.70 (7.89)	30.60 (4.81)	24.40 (6.72)
No	499 (95.96)	31.99 (6.07)	27.89 (5.04)	27.22 (4.82)
*t*		13.986	2.844	3.302
*P*		**≤0.001**	0.092	0.070

*Alcohol*				
Yes	38 (7.31)	29.92 (6.98)	29.13 (4.65)	27.08 (5.31)
No	482 (92.69)	32.00 (6.10)	27.85 (5.06)	27.17 (4.84)
*t*		4.016	2.287	0.013
*P*		**0.046**	0.131	0.908

*N* = 520. The bold values represent statistically significant *P* values (*P* < 0.05).

**Table 2:** The level of compassion satisfaction, burnout and secondary traumatic stress of new nurses.

Variable	Mean (SD)	Low level	Moderate level	High level
		*N* (%)	*N* (%)	*N* (%)
Compassion satisfaction	31.85 ± 6.18	30 (5.77)	457 (87.88)	33 (6.35)
Burnout	27.94 ± 5.04	76 (14.62)	439 (84.42)	5 (0.96)
Secondary traumatic stress	27.17 ± 4.87	86 (16.54)	431 (82.88)	3 (0.58)

*N* = 520.

**Table 3:** Multiple linear regression analyses of compassion satisfaction, burnout, and secondary traumatic stress.

Variable	*B*	SE	Beta	*t*	*P*
*Compassion satisfaction* ^*a*^					
(Constant)	13.396	3.541		3.783	≤0.001
Job satisfaction	3.415	0.310	0.444	11.002	≤0.001
Sleep hours per day	1.299	0.511	0.099	2.543	0.011
Smoking	−4.422	1.767	−0.098	−2.503	0.013
Workplace violence experience	−1.022	0.503	−0.081	−2.030	0.043

*Burnout* ^*b*^					
(Constant)	39.075	0.793		49.294	≤0.001
Job satisfaction	−3.606	0.219	−0.575	−16.482	≤0.001
Sleep hours per day	−1.418	0.375	−0.133	−3.779	≤0.001
Work hours per day	0.931	0.382	0.086	2.438	0.015

*Secondary traumatic stress* ^*c*^					
(Constant)	28.233	0.739		38.225	≤0.001
Workplace violence experience	1.222	0.436	0.122	2.806	0.005
Work hours per day	0.927	0.456	0.089	2.031	0.043

^a^ *F* = 37.799, *P* ≤ 0.001, and adjusted *R*^*2*^ = 0.221; ^b^*F* = 108.167, *P* ≤ 0.001, and adjusted *R*^*2*^ = 0.383; ^c^*F* = 6.622, *P* ≤ 0.001, and adjusted *R*^*2*^ = 0.021. *N* = 520.

## Data Availability

The data used to support the findings of this study are available from the corresponding authors upon reasonable request.
